# The efficacy and safety of dupilumab therapy in pediatric patients with refractory eosinophilic duodenal bulb ulcer: a retrospective cohort study

**DOI:** 10.3389/fimmu.2026.1953390

**Published:** 2026-10-27

**Authors:** Zhiheng Huang, Ping Li, Ying Zhou, Yangyang Ma, Shengnan Wang, Cuifang Zheng, Lin Wang, Zhinong Jiang, Yuhuan Wang, Ying Huang

**Affiliations:** 1 Department of Gastroenterology, Children’s Hospital of Fudan University, National Children’s Medical Center, Shanghai, China; 2 Department of Pathology, Children’s Hospital of Fudan University, National Children’s Medical Center, Shanghai, China; 3 Department of Pathology, Sir Run Run Shaw Hospital, Zhejiang University School of Medicine, Hangzhou, China

**Keywords:** biologic therapy, duodenal ulcer, dupilumab, eosinophilic gastrointestinal disease, pediatric

## Abstract

**Background:**

Refractory eosinophilic duodenal bulb ulcer (REDU) is a rare and poorly characterized subtype of non–eosinophilic esophagitis eosinophilic gastrointestinal disease (non-EoE EGID). Standard therapies are often ineffective, and evidence for biologic treatment is limited.

**Objective:**

This study aims to evaluate the efficacy and safety of dupilumab in pediatric patients with REDU.

**Methods:**

This retrospective cohort study included 13 pediatric patients with histologically confirmed REDU treated with dupilumab for 12 weeks between January and December 2025, followed by a 24-week extension period. Primary outcomes were endoscopic healing and histological remission. Secondary outcomes included symptom improvement, laboratory parameters, growth indices, and quality of life (EGGE-QOL). An exploratory cross-sectional transcriptomic analysis compared pretreatment REDU biopsies with healthy-control biopsies.

**Results:**

All patients achieved marked clinical improvement after 12 weeks of treatment. The median duodenal eosinophil count decreased significantly from 95 (IQR 72.5–100.0) to 3.5 (1.5–10.0) cells/HPF (P < 0.001), with histological remission achieved in all cases. Peripheral eosinophil counts and hemoglobin levels were improved significantly (both P < 0.05). Endoscopic mucosal healing was observed in all patients, with 11 achieving scar-stage healing. EGGE-QOL scores improved significantly (61.85 ± 16.90 vs 32.31 ± 18.85, *P* < 0.001). Exploratory RNA sequencing of pretreatment REDU biopsies versus healthy-control biopsies identified 837 genes meeting the nominal screening criteria, including 472 with higher and 365 with lower expression in pretreatment REDU biopsies. The top-ranked functional annotations were related to immune responses and cytokine signaling.

**Conclusions:**

Dupilumab demonstrated promising efficacy and acceptable safety in pediatric REDU, with consistent clinical, histological, and molecular improvements. These findings support further prospective studies to validate its long-term efficacy in non-EoE EGIDs.

## 1 Introduction

Eosinophilic gastrointestinal diseases (EGIDs) comprise a heterogeneous group of disorders characterized by eosinophilic infiltration of the gastrointestinal tract. EGIDs are broadly classified into eosinophilic esophagitis (EoE), in which inflammation is confined to the esophagus, and non-EoE EGIDs, which involve other segments of the gastrointestinal tract (1, 2). While EoE has been extensively investigated in both clinical and translational research, non-EoE EGIDs remain relatively understudied because of their rarity (3). Non-EoE EGIDs present with heterogeneous clinical manifestations, including abdominal pain, intestinal cramping, diarrhea, nausea, vomiting, abdominal distension, anorexia with weight loss, and gastrointestinal bleeding (4).

Eosinophilic duodenal ulcer is a rare subtype of non-EoE EGIDs. In particular, cases predominantly involving the duodenal bulb remain poorly characterized in the literature (5). Management of refractory eosinophilic duodenal bulb ulcer (REDU) represents a major clinical challenge, as no standardized treatment strategy has been established. Therapeutic approaches, including elimination diets, proton pump inhibitors (PPIs), and corticosteroids, have been used in these patients; however, treatment responses are often suboptimal (6).

Type 2 immune responses play a central role in the pathogenesis of EGIDs. Interleukin-4 (IL-4) and interleukin-13 (IL-13), key effector cytokines of type 2 immunity, are predominantly produced by T helper 2 (Th2) cells (7). These cytokines share a common receptor subunit, IL-4 receptor alpha (IL-4Rα). IL-13 promotes the recruitment and accumulation of eosinophils within the gastrointestinal mucosa (8). Activated eosinophils subsequently release cytotoxic granule proteins, including major basic protein and eosinophil peroxidase, which directly damage intestinal epithelial cells and result in epithelial erosion and ulcer formation (9).

Dupilumab (DUP) is a fully human monoclonal antibody targeting IL-4Rα, thereby blocking signaling pathways mediated by both IL-4 and IL-13 (10). DUP has recently been approved for the treatment of EoE in adults, adolescents, and children (11). Although several case reports have described successful treatment with DUP in children with eosinophilic duodenal bulbar ulcers (12, 13), data regarding DUP use in pediatric patients with REDU remain extremely limited, and its therapeutic efficacy and safety profile in this population remain unclear.

Therefore, the present study aimed to evaluate the efficacy and safety of DUP in pediatric REDU, with a focus on endoscopic healing, histological remission, and patient-reported clinical outcomes.

## 2 Materials and methods

### 2.1 Study design and participants

This study was approved by the Ethics Committee of the Children’s Hospital of Fudan University, Shanghai, China (IRB No. 2024-248) and was registered at ClinicalTrials.gov (NCT07257835). All procedures were conducted in accordance with the Declaration of Helsinki and relevant institutional guidelines and regulations. Written informed consent was obtained from the parents or legal guardians of all participants before enrollment.

Pediatric patients with REDU who received DUP treatment were retrospectively enrolled between January 1 and December 30, 2025.

### 2.2 Eligibility criteria

Patients were eligible if they met the following criteria: (1) histopathological evidence of a peak eosinophil count ≥50 cells per high-power field (eos/HPF) in duodenal bulb biopsy specimens; (4) and (2) an inadequate response to prior treatment with proton pump inhibitors (PPIs), corticosteroids, or immunosuppressive agents, or concerns about potential adverse effects associated with long-term use of these therapies.

The exclusion criteria for REDU: (1) Helicobacter pylori (HP) infection was excluded based on a negative clinical history and a negative ¹³C urea breath test (¹³C-UBT); (2) medication-related mucosal injury, particularly that associated with non-steroidal anti-inflammatory drugs, was excluded by detailed review of medication history; (3) inflammatory bowel disease and celiac disease were excluded through an integrated assessment clinical history, laboratory findings, endoscopic evaluation, and histopathological examination of mucosal biopsy specimens;(4) parasitic infections were excluded by targeted history taking and stool parasitological testing; and (5) systemic eosinophilic disorders, including hypereosinophilic syndrome, eosinophilic granulomatosis with polyangiitis, and other systemic eosinophilic conditions, were excluded through a multidisciplinary consultation.

Definitions were as follows: (1) REDU was defined as a duodenal bulb ulcer that failed to heal at the first follow-up endoscopy despite standard medical therapy, or that relapsed after initial healing during subsequent follow-up, with prominent eosinophilic infiltration confirmed histopathologically; (2) endoscopic healing was defined as complete mucosal healing of duodenal bulb lesions according to the endoscopic staging system for peptic ulcer; (3) histopathological remission was defined as improvement in mucosal histopathology after treatment, with ≤15 eos/HPF in duodenal bulb biopsy specimens and <6 eos/HPF in gastric biopsy specimens; (4) treatment response was defined as preliminary therapeutic improvement after intervention, encompassing symptomatic, endoscopic, and histological improvement.

### 2.3 Treatment protocol

This study protocol comprises a 12-week short-term observational period followed by a 24-week extended follow-up period. During the initial treatment phase, patients received weight-based DUP according to previously published studies (11, 14): 200 mg every 2 weeks for patients weighing ≥15 kg to <30 kg; 300 mg every 2 weeks for those weighing ≥30 kg to <60 kg; and 300 mg once weekly for those weighing ≥60 kg. All patients received concomitant PPI therapy (omeprazole, 0.5-1mg/kg/day or lansoprazole, 0.5mg/kg/day) during the 12-week treatment period, followed by gradual PPI tapering over the subsequent 24 weeks.

### 2.4 Endoscopic assessment

Endoscopic evaluations were performed at three time points: before DUP initiation and at 12 and 36 weeks after treatment initiation. We used the endoscopic staging system for peptic ulcer, in which duodenal bulbar ulcers are classified as active, healing, or scarring stages (15). Ulcer size, depth, healing status, and risk of complications were systematically assessed.

Giant duodenal bulb ulcers (GDBUs) were defined as ulcers with a maximal diameter of ≥2 cm on endoscopic examination.

### 2.5 Histological assessment

Eosinophils were counted in five non-overlapping high-power fields by two experienced gastrointestinal pathologists who were blinded to the clinical data. The peak eosinophil count was recorded for analysis.

### 2.6 Outcome measures

The primary outcomes were endoscopic ulcer healing and histological remission of eosinophilic infiltration.

Secondary outcomes included improvement in clinical symptoms, peripheral blood eosinophil counts, serum total IgE and allergen-specific IgE levels, and growth parameters and quality of life.

Nutritional status was assessed using Centers for Disease Control and Prevention (CDC) growth charts. A body mass index (BMI)-for-age below the 5th percentile was defined as underweight.

Safety outcomes included the incidence of adverse events and serious adverse events during DUP treatment.

### 2.7 Exploratory transcriptomic sequencing and bioinformatics analysis

Total RNA was extracted using TRIzol^®^ reagent. Library preparation and RNA sequencing were performed by Majorbio Co., Ltd. (Shanghai, China) using the Illumina NovaSeq 6000 platform (Illumina, San Diego, CA, USA).

The transcriptomic analysis was restricted to an exploratory cross-sectional comparison between pretreatment REDU biopsies (n = 7) and independent healthy-control biopsies (n = 4), to characterize disease-associated mucosal gene expression patterns for future investigation. Differential-expression analysis was performed using DESeq2, with the pretreatment REDU and healthy-control samples treated as independent groups.

Candidate genes were selected using the exploratory screening criteria of nominal *P* ≤ 0.05 and |log_2_ fold change| ≥ 1. The fold-change threshold was used to prioritize estimated expression differences of at least twofold, whereas the nominal *P*-value threshold served only as an initial exploratory screening criterion. Because the nominal *P*-value criterion does not control the false discovery rate(FDR) across genes, genes meeting these criteria were treated as exploratory candidates rather than as FDR-significant findings.

Gene Ontology(GO) enrichment analysis was performed using GOATOOLS, and Kyoto Encyclopedia of Genes and Genomes (KEGG) pathway enrichment was assessed by over-representation analysis implemented in Python and the Majorbio Cloud Platform. Multiple-testing correction was applied to the enrichment analyses, and the adjusted *P* values are displayed. The functional-enrichment analyses were used for hypothesis generation and were interpreted as associations with the exploratory candidate gene set rather than evidence of pathway activation or direct mechanistic validation.

### 2.8 EGID quality of life and symptom assessment

Health-related quality of life was assessed using the Eosinophilic Gastritis and Gastroenteritis Quality of Life (EGGE-QOL) questionnaire, a 20-item patient-reported outcome measure developed from qualitative patient interviews (16).

The assessed symptoms included abdominal pain, nausea, vomiting, early satiety, diarrhea, and bloating. Symptom burden was evaluated in terms of frequency, severity, and duration using a 6-point Likert scale. Higher scores indicated greater symptom severity and disease burden.

### 2.9 Statistical analysis

Continuous variables were expressed as mean ± standard deviation (SD) or median with interquartile range (IQR), as appropriate according to data distribution. Comparisons between paired observations were performed using the paired Student’s t-test or Wilcoxon signed-rank test. Categorical variables were compared using the chi-square test or Fisher’s exact test when appropriate.

All statistical analyses were conducted using SPSS version 22.0 (IBM SPSS Statistics, Chicago, IL, USA). A two-sided P value <0.05 was considered statistically significant.

## 3 Results

### 3.1 The baseline data

Of the 32 children diagnosed with REDU, 14 were treated with DUP, and the remaining 18 patients did not receive DUP therapy for the following reasons: parental refusal of treatment due to financial burden, well-controlled disease status with PPI monotherapy, or unwillingness of the families to complete regular follow-up visits at our center.

Of the 14 children treated with DUP, none had received prior biologic therapy. All patients received DUP in combination with PPI therapy during the 12 weeks study period. One patient was excluded because of non-adherence to the DUP therapy regimen owing to financial difficulties. Consequently, 13 patients were included in the final analysis. **Figure 1** illustrates the patient screening and enrollment flow diagram of the present study.

**Figure 1:**
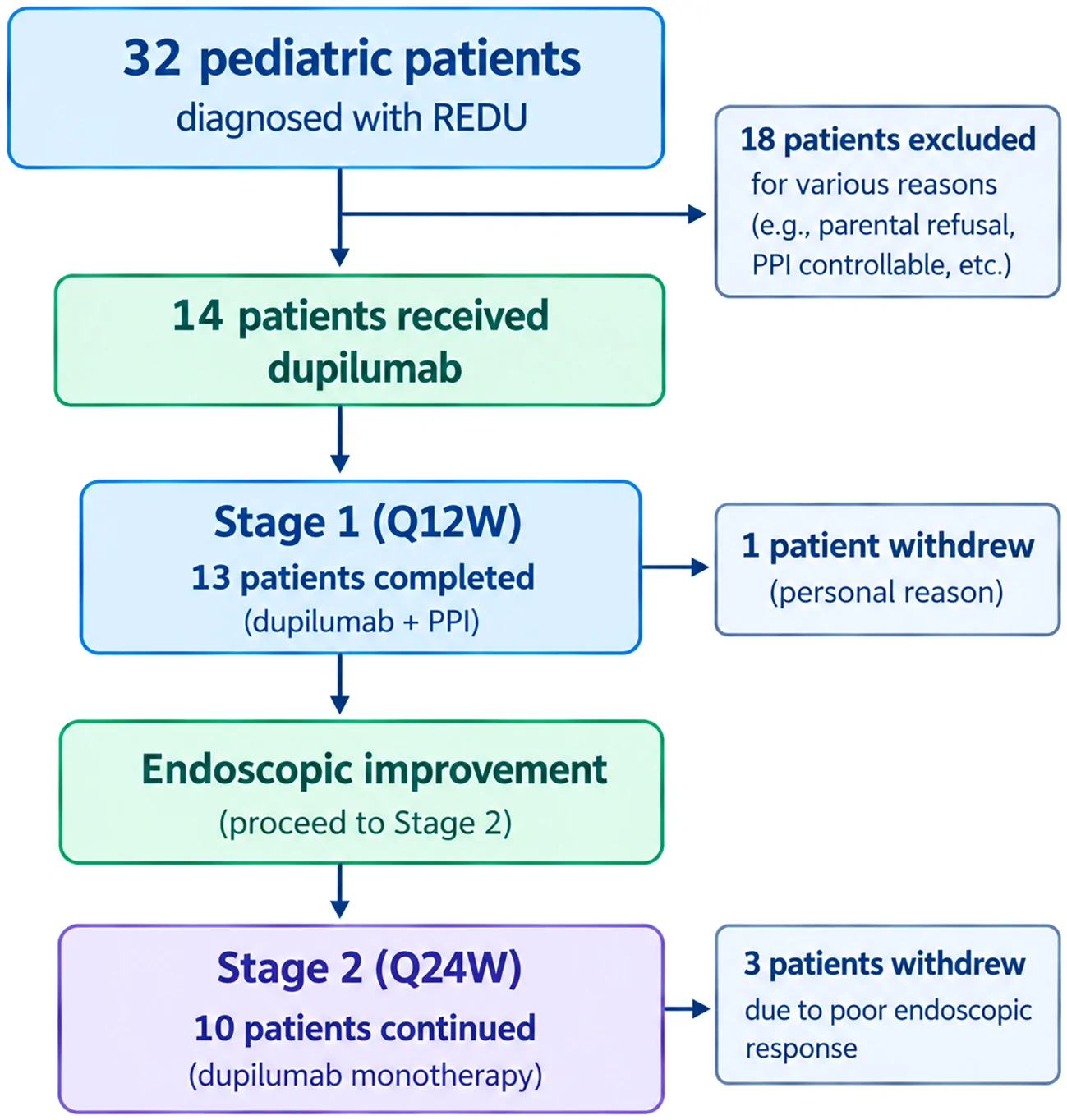
Flow diagram of patient screening and enrollment for dupilumab treatment in patients with refractory eosinophilic duodenal ulcer (REDU).

The mean age at symptom onset was 8.7 ± 2.9 years, and the mean age at admission was 10.4 ± 3.0 years. The mean body weight at admission was 35.6 ± 13.9 kg. Detailed baseline characteristics are summarized in **Table 1**. Four children had atopic comorbidities and did not receive any form of immunotherapy.

**Table 1:** Individual characteristics and treatment data for patients with REDU during the 12-week treatment period.

No.	Age/Sex	Main symptom	Atopic comorbidity	Prior treatment	Concomitant therapy during Dupilumab	Dupilumab usage
1	12/F	Abdominal pain	Rhinitis	Omeprazole	Omeprazole for 12 weeks	300mg, Q2W
2	8/M	Abdominal pain	Rhinitis	Omeprazole +CS+MTX	Omeprazole for 12 weeks for 5 weeks	200mg, Q2W
3	10/M	Abdominal pain	–	Omeprazole	Omeprazole for 12 weeks	300mg, Q2W
4	10/M	Abdominal pain	–	Omeprazole +CS+AZA	Omeprazole for 12 weeks +CS for 4 weeks	300mg, Q2W
5	12/M	Abdominal pain	–	Omeprazole +CS	Omeprazole for 12 weeks	300mg, Q2W
6	11/M	Hematemesis	Conjunctivitis	Omeprazole +CS+AZA	Omeprazole for 12 weeks +AZA for 3 weeks	300mg, Q2W
7	11/M	Abdominal pain	–	Omeprazole +AZA	Omeprazole for 12 weeks +AZA for 8 weeks	300mg, Q2W
8	11/M	Abdominal pain	–	Omeprazole	Omeprazole for 12 weeks	300mg, Q2W
9	16/M	DBU	–	Omeprazole +AZA+CS	Omeprazole for 12 weeks +AZA for 9 weeks	300mg, Q2W
10	14/F	Abdominal pain	Rhinitis	Omeprazole +AZA	Omeprazole for 12 weeks +AZA for 16 weeks	300mg, QW
11	6/M	Abdominal pain	–	Omeprazole +CS	Omeprazole for 12 weeks +CS for 11 weeks	200mg, Q2W
12	5/F	Abdominal pain	–	Omeprazole +CS	Omeprazole for 12 weeks	200mg, Q2W
13	9/F	Abdominal pain	–	Lansoprazole	Lansoprazole for 12 weeks	300mg, Q2W

AZA, azathioprine; CS, corticosteroid; DBU, duodenal bulb ulcer; F, female; M, male; MTX, methotrexate; QW, once weekly; Q2W, once every 2 weeks; REDU, refractory eosinophilic duodenal bulb ulcer.

Notably, seven of the 13 patients had a history of HP infection and subsequently developed REDU despite successful eradication therapy, as confirmed by negative repeat C^13^ urea breath tests. The median time interval between successful HP and REDU diagnosis was 9.0 [4.0–12.0] months, with no evidence of HP infection at the time of treatment.

### 3.2 Improvement in clinical symptoms and laboratory parameters following 12 weeks of DUP therapy

All patients experienced clinical improvement following DUP therapy. Abdominal pain, the most common presenting symptom, decreased from 11/13 (84.6%) at baseline to 2/13 (15.4%) after treatment (**Table 2**). In addition, atopic comorbidities showed marked improvement during the therapy period.

**Table 2:** Clinical symptoms, laboratory parameters, and growth indicators before and after dupilumab treatment.

Parameter	Before DUP treatment	After DUP treatment	*P* value
Abdominal pain, n (%)	11(84.62)	2(15.38)	**0.001**
Anemia, n (%)	7 (46.15)	0 (0)	**0.002**
Vomiting, n (%)	2 (15.38)	0 (0)	0.086
Dizziness, n (%)	1(7.69)	0 (0)	0.317
Weight loss, n (%)	2(15.38)	1(7.69)	0.539
Fatigue, n (%)	5(38.46)	0 (0)	**0.013**
BW (kg)	35.58 ± 13.89	40.34 ± 13.56	0.492
Height (cm)	145.61 ± 18.35	150.38 ± 18.61	0.517
BMI(kg/m^2^)	16.80 ± 3.42	17.35 ± 2.92	0.663
Hb (g/L)	113.92 ± 24.35	132.61 ± 12.32	**0.021**
EOS (cells/ul)	410[160,525]	190[45,240]	**0.033**
IgE (KU/L)	237.0[78.75,730.5]	175.2[79.50,451.0]	0.330
WBC(x10^9/L)	6.44 ± 1.87	5.59 ± 1.06	0.164
Albumin (g/L)	45.22 ± 6.72	44.17 ± 2.91	0.622
Prealbumin (mg/L)	222.68 ± 38.39	230.92 ± 37.74	0.594
EOS in duodenal bulb(cells/HPF)	95[72.5,100.0]	3.5[1.5,10]	**<0.001**
EOS in gastric antrum(cells/HPF)	2.0[0,80]	0.5[0,5]	0.24

BMI, body mass index; BW, body weight; EOS, eosinophil count; Hb, hemoglobin; HPF, high-power field; IgE, immunoglobulin E; WBC, white blood cell count. Bold values indicate statistically significant differences.

Laboratory assessments revealed significant increases in hemoglobin levels and significant reductions in peripheral eosinophil counts after treatment relative to baseline (both *P* < 0.05) (**Table 2**). Furthermore, at 12 weeks post-treatment, DUP was associated with substantial reductions in peripheral eosinophils (absolute change: −220 cells/μL; relative change: −53.7%; median difference, 95% CI: −345 to −95) and tissue eosinophils (absolute change: −91.5 percentage points; relative change: −96.3%; 95% CI: −98.2 to −78.6), as well as a marked increase in hemoglobin (absolute change: +18.69 g/dL; relative change: +16.4%; Cohen’s *d* = 0.77, 95% CI: 0.34–1.21).

Food allergen testing was positive in 11 patients, most commonly to cow’s milk protein (5/11), egg white (6/11), and egg yolk (4/11). Inhalant sensitization to house dust mites was detected in 11 patients. Following DUP treatment, patients self-reported marked improvements in food tolerance. Seven patients successfully tolerated previously restricted foods, including milk and eggs, without developing obvious allergic reactions.

### 3.3 Endoscopic outcomes

All patients underwent serial upper gastrointestinal endoscopic evaluation, with a mean of 7.92 ± 4.34 procedures per patient.

At baseline, all patients had active-stage duodenal bulb ulcers, and GDBUs were present in 9/13 (69.2%) patients.

After 12 weeks of DUP therapy, endoscopic improvement was observed in all patients. Eleven patients entered the scar stage, while the other two entered the healing stage. (**Figures 2A–D**). Individual endoscopic responses over the 12-week course of DUP therapy are presented in **Supplementary Figures 1**, **2**.

**Figure 2:**
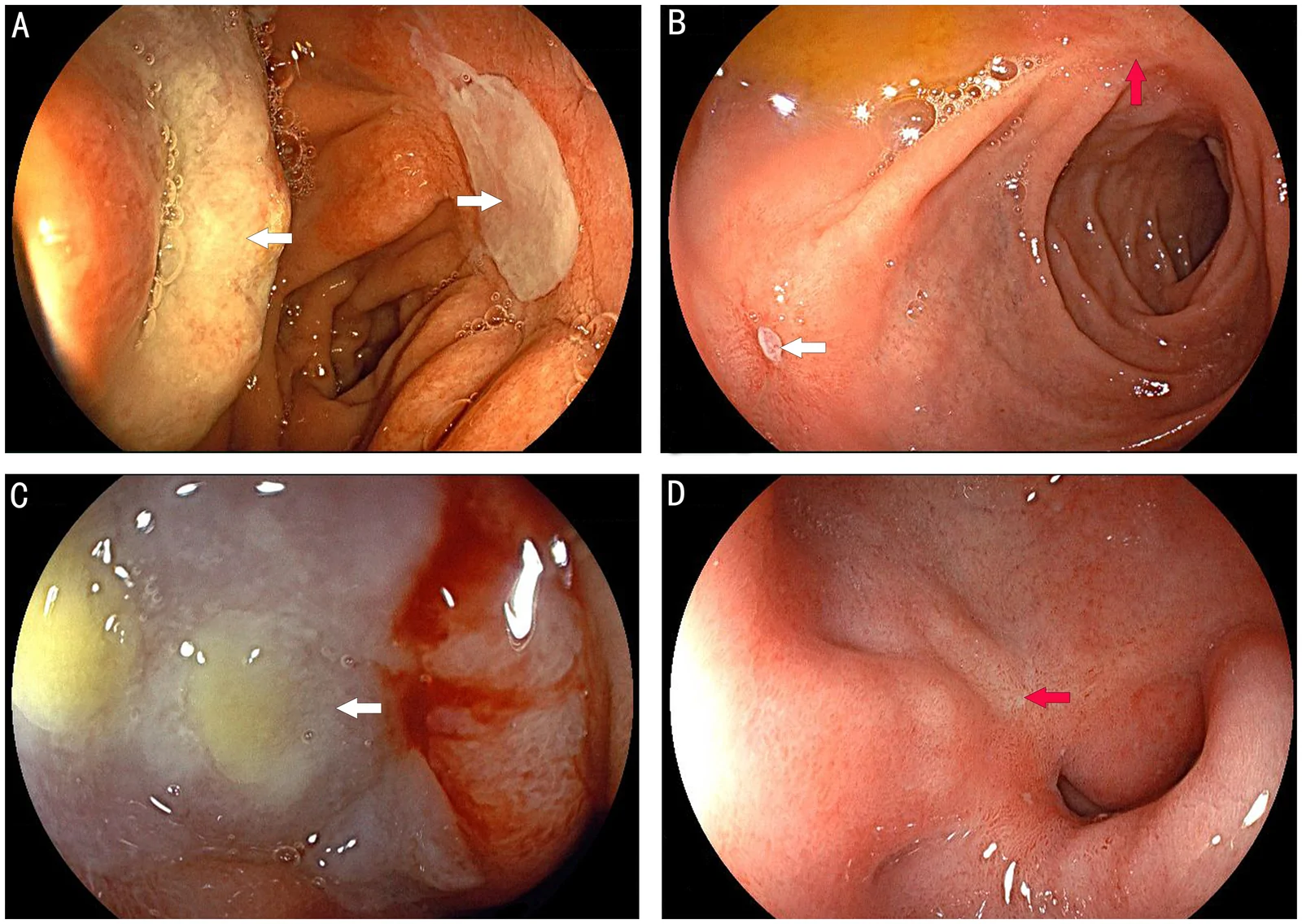
Endoscopic findings in the duodenal bulb before and after dupilumab treatment. **(A)** Case 1 before treatment, showing giant active stage kissing ulcers in the duodenal bulb: an approximately 2.5 × 3.0 cm ulcer on the anterior wall and an approximately 1.5 × 2.5 cm ulcer on the posterior wall (white arrow). **(B)** Case 1 after 12 weeks of dupilumab treatment, showing an approximately 0.3 × 0.5 cm ulcer on the anterior wall (white arrow) and scar formation on the posterior wall (red arrow). **(C)** Case 3 after Helicobacter pylori eradication, showing a giant active-stage postbulbar duodenal ulcer measuring approximately 1.5 × 4.0 cm (white arrow). **(D)** Case 3 after 12 weeks of dupilumab treatment, showing progression to the scar stage with duodenal bulb stenosis (red arrow).

Three patients had bulbar strictures at baseline. After 12 weeks of treatment, strictures resolved in two patients and improved in one patient. At week 12 following DUP initiation, six children developed bulbar strictures. Five of these patients had baseline postbulbar duodenal ulcers, which progressed to fibrotic strictures after 12-week therapy.

### 3.4 Histological findings

Histological examination was performed in all patients. Prior to treatment, duodenal bulb biopsies demonstrated marked eosinophilic infiltration within the lamina propria. Increased eosinophil infiltration in the gastric antrum was also observed in five patients.

Following DUP therapy, duodenal eosinophil counts significantly decreased from 95 (IQR:72.5–100.0) to 3.5 (IQR:1.5–10.0) cells/HPF (P<0.001) (**Figures 3A–E**). The paired changes in duodenal bulb eosinophil counts/HPF for individual patients before and after 12 weeks of DUP therapy are presented in **Figure 3F**. Although gastric antral eosinophil counts also decreased after treatment, the difference was not statistically significant.

**Figure 3:**
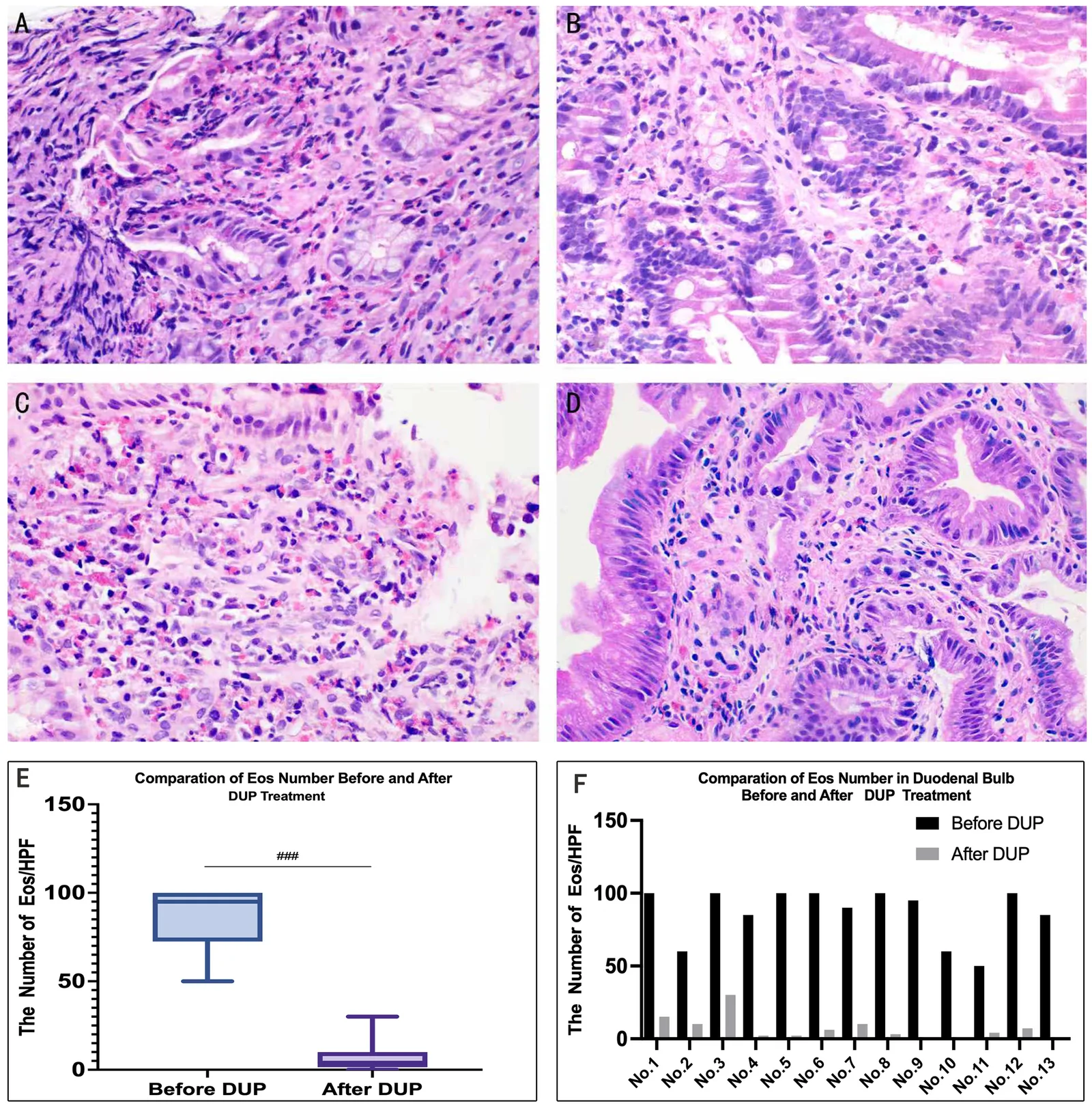
Histological response after 12 weeks of dupilumab treatment. **(A)** Case 1 before treatment, showing marked eosinophilic infiltration in the duodenal bulb (>100 eosinophils (EOS)/high-power field [HPF]). **(B)** Case 1 after treatment, showing a marked reduction in eosinophilic infiltration (15 EOS/HPF). **(C, D)** Case 3 showed a similar histopathological response (>100 vs. 30 EOS/HPF before and after treatment, respectively). **(E)** Box plot of duodenal bulb eosinophil counts before and after treatment in the 13 patients. **(F)** Individual patient data before and after dupilumab treatment.

### 3.5 Exploratory cross-sectional transcriptomic comparison

The exploratory transcriptomic analysis was restricted to the cross-sectional comparison between pretreatment REDU biopsies (n = 7) and independent healthy-control biopsies (n = 4). Using the exploratory screening criteria of nominal *P* ≤ 0.05 and |log2 fold change| ≥ 1, 837 genes met the screening criteria. Of these, 472 showed higher expression and 365 showed lower expression in pretreatment REDU biopsies relative to healthy controls. These genes were treated as exploratory candidates and should not be interpreted as FDR-significant differentially expressed genes.

GO over-representation analysis of the candidate gene set highlighted top-ranked terms related to immune response, extracellular space, response to external stimuli, defense response, cytokine-mediated signaling, and inflammatory response. The top-ranked KEGG annotations included cytokine–cytokine receptor interaction, IL-17 signaling, hematopoietic cell lineage, and chemokine signaling pathways (**Figure 4**). These functional annotations are hypothesis-generating and do not establish pathway activation or validated disease mechanisms. No inference regarding longitudinal transcriptomic changes or the molecular effects of dupilumab was made from this analysis.

**Figure 4:**
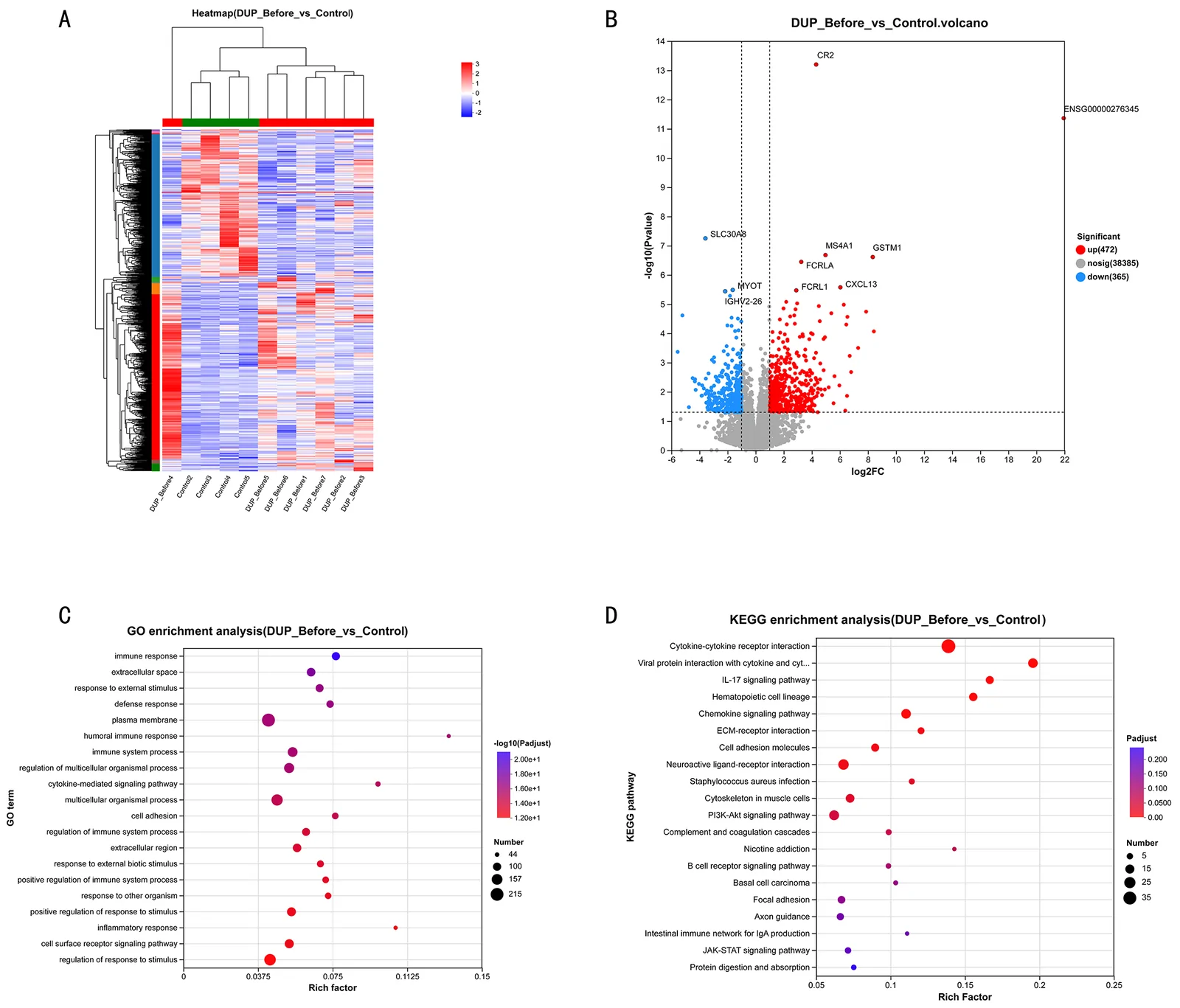
Exploratory cross-sectional transcriptomic comparison between pretreatment REDU biopsies and healthy-control biopsies. Pretreatment REDU biopsies (n=7) and independent healthy-control biopsies (n=4) were compared using DESeq2. Candidate genes were defined using the exploratory screening criteria of nominal *P* ≤ 0.05 and |log_2_ fold change| ≥ 1. **(A)** Hierarchically clustered heatmap of the exploratory candidate gene set. **(B)** Volcano plot showing 472 genes with higher expression and 365 genes with lower expression in pretreatment REDU biopsies relative to healthy controls. Positive log_2_ fold-change values indicate higher expression in pretreatment REDU biopsies, whereas negative values indicate lower expression. The vertical and horizontal dashed lines indicate |log_2_ fold change| = 1 and nominal *P* = 0.05, respectively. **(C)** GO over-representation analysis of the candidate gene set. **(D)** KEGG over-representation analysis of the candidate gene set. Dot size represents the number of mapped candidate genes, and color corresponds to the adjusted P-value scale shown in each panel. The displayed terms and pathways represent top-ranked functional annotations; not all displayed pathways necessarily met an adjusted P < 0.05 threshold. These analyses are exploratory and identify transcriptomic associations rather than validated disease mechanisms.

### 3.6 Follow-up outcomes

At a week 12, sustained clinical and endoscopic improvement was observed in all patients.

All seven patients previously treated with corticosteroids discontinued steroid therapy, and all six patients previously receiving immunosuppressive agents discontinued these medications. Nine of 13 patients (69.2%) discontinued PPI therapy, while the remaining four were undergoing gradual tapering with planned discontinuation.

Mean body weight, height, and BMI increased numerically during follow-up; however, these changes did not reach statistical significance due to the short follow-up duration (**Table 2**).

During the subsequent 24-week follow-up, 10 patients (76.9%) maintained healing of duodenal bulb ulcers after PPI discontinuation, with concurrent resolution of fibrotic strictures. Of the six children who developed bulbar strictures at week 12 of DUP treatment, strictures resolved in four and ulcer recurrence occurred in two following an additional 24-week course of DUP monotherapy.

Overall, three patients on continuous DUP monotherapy experienced ulcer recurrence complicated by anemia, prompting discontinuation of DUP at subsequent 24-week. Two of these three patients were among the six children with week-12-onset bulbar strictures. Of these recurrent cases, one was administered upadacitinib and one received high-dose PPI, with both still being followed up. The remaining patient turned to herbal therapy and PPI in another hospital and was lost to follow-up (See **Supplementary Table 1**; **Figure 4**).

### 3.7 EGID-QOL and symptom severity assessment

EGID-QOL improved significantly following DUP therapy at week 12. The frequency, severity, and duration of abdominal pain, the most prominent symptom in REDU, decreased significantly after treatment (all *P* < 0.05). Similarly, total EGGE-QOL scores improved from 61.85 ± 16.90 at baseline to 32.31 ± 18.85 post-treatment (*P* < 0.001) (**Tables 3**, **4**). The EGGE-QOL score showed an absolute reduction of −29.54 points and a relative reduction of −47.8% (Cohen’s d = 1.75, 95% CI: 1.02–2.48), reflecting a substantial decrease in disease burden and improvement in health-related quality of life.

**Table 3:** The symptom severity scale of patients before and after dupilumab treatment.

Item	Symptom frequency (*P* value)	Symptom severity (*P* value)	Symptom duration (*P* value)
Pain	0.006	0.006	0.007
Nausea	0.022	0.059	0.029
vomiting	0.025	0.075	0.029
Satiety	0.107	0.167	0.082
Diarrhea	0.308	0.154	0.154
Bloating	0.035	0.046	0.062

**Table 4:** EGGE-QOL scores before and after dupilumab (DUP) treatment (Bold values indicate statistically significant differences).

Item	Before-DUP	After-DUP	*P* value
1.Worry about many parts of life impacted	3.92 ± 0.95	2.38 ± 1.33	**0.002**
2.Stress at work or school	3.38 ± 1.45	1.54 ± 1.51	**0.004**
3.Hopelessness	2.46 ± 1.56	0.77 ± 1.17	**0.005**
4.Down, sad, or depressed	3.0 ± 1.47	0.66 ± 1.11	**<0.001**
5.Stressful finding medical providers	3.23 ± 1.42	1.38 ± 1.56	**0.004**
6.Social withdrawal	1.85 ± 1.14	1.0 ± 1.15	**0.073**
7.Reduced physical activity	3.24 ± 1.24	1.46 ± 1.33	**0.002**
8.Intimate relationships/sexual function impacted	1.62 ± 1.04	1.08 ± 1.44	0.286
9.Difficult to keep/find a job or finish school	2.08 ± 1.04	0.85 ± 1.28	0.13
10.People don’t understand condition	3.15 ± 1.34	1.46 ± 1.45	**0.05**
11. How I feel about managing EGID not improving over time	3.08 ± 1.19	0.77 ± 0.73	**<0.001**
12. Worry about diet	4.08 ± 1.19	2.30 ± 1.60	**0.04**
13. Body dissatisfaction	2.54 ± 1.56	1.31 ± 1.11	0.29
14.Anxiety about eating out	3.77 ± 1.24	1.54 ± 0.97	**0.039**
15.Important relationships have suffered	3.00 ± 1.83	1.08 ± 1.50	**0.077**
16.Worry about getting cancer or other serious diagnosis	1.54 ± 0.97	1.85 ± 1.52	0.544
17.Concerned about lack of medical options	4.15 ± 1.21	1.69 ± 1.80	**<0.001**
18.Worry about safety/long term medication effects	4.62 ± 0.51	3.38 ± 0.65	**<0.001**
19.Big increase in out-of-pocket costs	4.23 ± 1.09	3.08 ± 1.12	**0.014**
20.Food less enjoyable	2.92 ± 1.50	1.62 ± 1.50	**0.036**
**Total EGGE-QOL score**	**61.85 ± 16.90**	**32.31 ± 18.85**	**<0.001**

### 3.8 Safety

DUP was generally well tolerated. One patient experienced transient elevation of liver transaminases, which resolved spontaneously. Another patient developed a mild rash that resolved without intervention. No serious adverse events were reported during the study period.

## 4 Discussion

EGIDs are rare chronic inflammatory disorders characterized by eosinophilic infiltration of the gastrointestinal tract [12]. EGIDs can be triggered by a wide variety of factors, including incomplete eradication of HP, medications, and immunotherapy (17, 18). Although several children in our cohort had comorbidities, none of them had received immunotherapy. Therefore, other contributing factors may be responsible for the development of this disorder.

REDU represents a particularly uncommon and clinically challenging subtype of non-EoE EGIDs due to its recurrent course, resistance to conventional therapies, and lack of standardized treatment strategies. Deslandres et al. first reported a pediatric case of eosinophilic gastroenteritis complicated by perforated duodenal ulcer, highlighting the potential severity of eosinophilic inflammation in the duodenum [13]. Although isolated cases of eosinophilic duodenal ulcers have been reported, the clinical characteristics and optimal management of REDU remain poorly defined [2]. In a previous multicenter study, we described the clinical course of REDU following HP eradication [5]. Building on these observations, the present study represents, to our knowledge, one of the largest single-center pediatric cohorts evaluating DUP in REDU, and among the first reports from China describing its clinical efficacy, histological response, and exploratory transcriptomic changes in this disease.

The primary therapeutic goals in non-EoE EGIDs are symptom control, suppression of mucosal inflammation, induction of tissue healing, and prevention of long-term complications (19). However, currently available treatments, including elimination diets, PPIs, corticosteroids, and immunosuppressive agents, often yield incomplete responses and may be associated with significant adverse effects, particularly in children requiring prolonged therapy (20). In our cohort, all patients had previously failed conventional treatment and were therefore classified as having refractory disease. Following DUP concomitant therapy, all patients demonstrated marked clinical improvement, accompanied by significant reductions in peripheral eosinophil counts and substantial endoscopic and histological healing.

The therapeutic efficacy of DUP is biologically plausible given the central role of type 2 immune responses in EGID pathogenesis. IL-4 and IL-13 are key cytokines involved in eosinophil recruitment, activation, survival, and tissue remodeling within the gastrointestinal tract. By targeting the IL-4Rα subunit, DUP simultaneously blocks both IL-4- and IL-13-mediated signaling pathways, thereby suppressing eosinophilic inflammation and promoting mucosal healing (21). Consistent with this mechanism, our study demonstrated a dramatic reduction in mucosal eosinophil infiltration of duodenal bulb, with median eosinophil counts decreasing from 95 cells/HPF before treatment to 3.5 cells/HPF after treatment. These findings support the hypothesis that dual blockade of IL-4 and IL-13 signaling may be particularly effective in controlling type 2 inflammation in REDU.

Our findings are consistent with previous reports. Patel et al. (22) described marked reductions in gastrointestinal eosinophilia in pediatric patients treated with DUP, suggesting potential efficacy in non-EoE EGIDs. Braun et al. (23) reported pediatric cases of DUP use in eosinophilic gastrointestinal disorders with extraesophageal involvement. Tsuge et al. (13) reported a pediatric case of eosinophilic duodenal ulcer triggered by egg allergy achieving complete remission after DUP therapy. Similarly, Sánchez-Ramírez et al. (24) reported successful DUP therapy of severe eosinophilic gastroenteritis complicated by eosinophilic ascites in a 12-year-old patient. Visaggi et al. (25) demonstrated that DUP can induce and maintain remission in eosinophilic gastrointestinal disorders with concomitant eosinophilic esophagitis. Recently, Piccirillo et al. (26) described DUP as rescue therapy in steroid-dependent eosinophilic gastritis refractory to elimination diet. Compared with these isolated case reports and small series, our study provides more comprehensive evidence of consistent clinical, endoscopic, and histological responses in a larger pediatric REDU cohort.

One of the most notable findings of this study was the remarkable endoscopic healing observed after DUP treatment. Prior to treatment, all patients had active duodenal bulb ulcers, and nearly 70% met the criteria for giant duodenal bulb ulcers. After only 12 weeks of concomitant PPI treatment, all patients demonstrated endoscopic improvement. These observations indicate that PPIs promote active ulcer healing and potentiate the therapeutic efficacy of dupilumab through dual mechanisms: not only by suppressing gastric acid secretion, but also by exerting direct anti-inflammatory effects within the gastrointestinal mucosa. This local anti-inflammatory activity may reduce eosinophilic infiltration, thereby alleviate tissue injury and create a mucosal microenvironment favorable for dupilumab-mediated remission (27, 28). These findings suggest that eosinophilic inflammation may play a direct role in ulcer persistence and impaired mucosal repair. Once eosinophilic inflammation is effectively controlled, ulcer healing can occur rapidly even in patients with long-standing refractory disease. Similar fibrostenotic complications have been described in eosinophilic esophagitis (22). This finding suggests that chronic eosinophilic inflammation may induce tissue remodeling and fibrosis before treatment initiation. Interestingly, long-term DUP treatment can reduce the development of fibrotic strictures in eosinophilic esophagitis (22, 29, 30). In the present study, compared with 12-week DUP therapy, 36-week led to marked improvement of duodenal bulbar strictures in the majority of patients. Therefore, earlier diagnosis and timely initiation of effective DUP therapy may be critical for preventing structural complications in EGIDs.

Transcriptomic analysis provided exploratory molecular context for the observed clinical and histological response (28). Previous studies have demonstrated increased expression of Th2-associated cytokines, including IL-4, IL-5, IL-13, and eotaxin-3, in the gastrointestinal mucosa of patients with non-EoE EGIDs (31). Consistent with the immune-mediated nature of these disorders, our exploratory analysis suggested that the transcriptional alterations observed in pretreatment REDU mucosa were predominantly associated with immune and inflammatory processes, with enrichment involving cytokine- and chemokine-related signaling pathways. Notably, pathways related to cytokine–cytokine receptor interactions and IL-17 signaling were among the prominent signals, suggesting that the mucosal inflammatory landscape of REDU may extend beyond a purely Th2-associated response and involve broader immune-network perturbations. However, these transcriptomic findings should be interpreted as hypothesis-generating rather than mechanistic evidence. Given the small RNA-seq sample size, nominal *P*-value-based screening, and bulk-tissue design, the observed differences cannot establish a molecular response to DUP or a drug-specific mechanism of action. Validation in larger independent cohorts, together with appropriately modeled longitudinal transcriptomic studies, will be required to define the molecular features of REDU and determine how they change during treatment.

In addition to objective clinical and histological improvements, DUP significantly improved patient-reported outcomes. The EGGE-QOL instrument captures multiple dimensions of disease burden, including psychological well-being, daily functioning, medical burden, and dietary restriction (32). Significant improvement across these domains indicates that DUP not only alleviated gastrointestinal symptoms but also substantially reduced the overall impact of disease on patients’ and families’ daily lives.

Several limitations should be acknowledged. First, this was a single-center retrospective study with a relatively small sample size and no untreated or active comparator group, reflecting the rarity of REDU. Second, all patients received concomitant PPI therapy, which may confound the attribution of treatment effects. During the subsequent 24-week follow-up period, 23.1% (3/13) of patients experienced duodenal bulb ulcer recurrence with DUP monotherapy. This observation has not been previously reported in the available literature. We hypothesize that IL-4/IL-13 blockade alone may be insufficient in a subset of patients and that additional pathogenic pathways may contribute to disease persistence or recurrence (33). Further investigations into novel pathogenic mechanisms and alternative therapeutic strategies are warranted in future studies (34). Third, our endoscopic evaluation carried potential assessment bias. Endoscopic procedures and lesion assessments were not performed and reviewed consistently by a single endoscopist. In addition, the investigators were unblinded to patients’ treatment allocation during endoscopic interpretation. Both factors constituted potential sources of bias in ulcer staging. Fourth, transcriptomic analysis was performed in a limited subset of patients and cannot distinguish between true gene regulation and changes in cellular composition. Finally, although DUP demonstrated an acceptable safety profile in this cohort, larger studies with longer follow-up are required to confirm its long-term safety in pediatric patients.

In conclusion, DUP therapy was associated with promising clinical, endoscopic, histological, and patient-reported improvements in pediatric patients with REDU, with an acceptable safety profile. These findings support DUP as a potential therapeutic option for this rare and difficult-to-treat condition and provide a rationale for future multicenter prospective studies aimed at optimizing treatment strategies, validating molecular biomarkers, and evaluating long-term outcomes.

## Data Availability

Publicly available datasets were analyzed in this study. This data can be found here: NCBI BioProject, accession number PRJNA1512062.

## References

[1] Soni M Peterson KA Uchida AM . Eosinophilic gastrointestinal disease. JAMA. (2024) 331:1407–8. doi: 10.1001/jama.2024.2143 38526463

[2] Visaggi P Dellon ES . Epidemiology, natural history, and treatment of eosinophilic gastrointestinal diseases. Gastroenterology. (2026) 170:476–94. doi: 10.1053/j.gastro.2025.09.030 41288538

[3] Lam AY Gonsalves N . Eosinophils beyond the esophagus: A review of non-Eoe eosinophilic gastrointestinal diseases. Gastroenterol Hepatol (N Y). (2025) 21:511–9. PMC12397780 40894984

[4] Papadopoulou A Amil-Dias J Auth MK Chehade M Collins MH Gupta SK . Joint Espghan/Naspghan guidelines on childhood eosinophilic gastrointestinal disorders beyond eosinophilic esophagitis. J Pediatr Gastroenterol Nutr. (2024) 78:122–52. doi: 10.1097/MPG.0000000000003877 38291684

[5] Fujita Y Tominaga K Ishida K Masuyama H Yoshihara S . Proton pump inhibitor to treat an eosinophilic duodenal ulcer with esophageal involvement: A pediatric case. Tohoku J Exp Med. (2022) 257:309–13. doi: 10.1620/tjem.2022.J045 35661048

[6] Huang Z Li P Zhou Y Ji M Ma Y Meng Y . Refractory eosinophilic duodenal bulb ulcer associated with Helicobacter pylori eradication in children: A multicenter study. Sci Rep. (2026) 16:6570. doi: 10.1038/s41598-026-37351-y 41611857 PMC12909989

[7] Ben-Baruch Morgenstern N Shoda T Rochman Y Caldwell JM Collins MH Mukkada V . Local type 2 immunity in eosinophilic gastritis. J Allergy Clin Immunol. (2023) 152:136–44. doi: 10.1016/j.jaci.2023.01.021 36754294 PMC10330288

[8] Batkhurel K Prasad S Potter M Martin JH Keely S Talley NJ . Therapies targeting mucosal eosinophils in eosinophilic gut diseases from esophagus to colon. J Gastroenterol Hepatol. (2026) 41:606–24. doi: 10.1111/jgh.70221 41510895

[9] Jacobs I Ceulemans M Wauters L Breynaert C Vermeire S Verstockt B . Role of eosinophils in intestinal inflammation and fibrosis in inflammatory bowel disease: An overlooked villain? Front Immunol. (2021) 12:754413. doi: 10.3389/fimmu.2021.754413 34737752 PMC8560962

[10] Dellon ES Rothenberg ME Collins MH Hirano I Chehade M Bredenoord AJ . Dupilumab in adults and adolescents with eosinophilic esophagitis. N Engl J Med. (2022) 387:2317–30. doi: 10.1056/NEJMoa2205982 36546624

[11] Chehade M Dellon ES Spergel JM Collins MH Rothenberg ME Pesek RD . Dupilumab for eosinophilic esophagitis in patients 1 to 11 years of age. N Engl J Med. (2024) 390:2239–51. doi: 10.1056/NEJMoa2312282 38924731

[12] Patel N Goyal A Thaker A Troendle D Parrish C . A case series on the use of dupilumab for treatment of refractory eosinophilic gastrointestinal disorders. J Pediatr Gastroenterol Nutr. (2022) 75:192–5. doi: 10.1097/MPG.0000000000003512 35666881

[13] Tsuge M Shigehara K Uda K Kawano S Iwamuro M Saito Y . Successful use of dupilumab for egg-induced eosinophilic gastroenteritis with duodenal ulcer: A pediatric case report and review of literature. Allergy Asthma Clin Immunol. (2023) 19:103. doi: 10.1186/s13223-023-00859-3 38053199 PMC10698892

[14] Sia T Bacchus L Tanaka R Khuda R Mallik S Leung J . Dupilumab can induce remission of eosinophilic gastritis and duodenitis: A retrospective case series. Clin Transl Gastroenterol. (2024) 15:e00646. doi: 10.14309/ctg.0000000000000646 37753954 PMC10811686

[15] Sakita T Oguro Y Takasu S Fukutomi H Miwa T . Observations on the healing of ulcerations in early gastric cancer. The life cycle of the malignant ulcer. Gastroenterology. (1971) 60:835–9. doi: 10.1016/s0016-5085(71)80082-3 5581326

[16] Taft TH Hirano I Gonsalves N . Initial validation of the eosinophilic gastritis and gastroenteritis quality of life scale. Gastro Hep Adv. (2025) 4:100627. doi: 10.1016/j.gastha.2025.100627 40655160 PMC12246544

[17] Rossi CM Terreehorst I Apostolidou E Votto M Bakirtas A Cianferroni A . A systematic review and meta-analysis on the induction of confirmed eosinophilic esophagitis as a side effect of allergen immunotherapy: An Eaaci task force report. Allergy. (2026) 81:1024–38. doi: 10.1111/all.70183 PMC13040641 41403138

[18] Gutierrez-Ramirez L Garcia-Dionisio SL Feo-Ortega S Gonzalez-Cervera J Tejera-Munoz A Lucendo AJ . The association between Helicobacter pylori infection and eosinophilic esophagitis: Systematic review and meta-analysis. Helicobacter. (2025) 30:e70038. doi: 10.1111/hel.70038 40249184

[19] Lenti MV Rossi CM Dellon ES Lucendo AJ Sawada A Cianferoni A . Eosinophilic gastrointestinal diseases. Nat Rev Dis Primers. (2026) 12(1):47. doi: 10.1038/s41572-026-00723-9 42493505

[20] Dickerson A Dellon ES Aceves SS . Future of therapy and monitoring for eosinophilic esophagitis and eosinophilic gastrointestinal diseases. Ann Allergy Asthma Immunol. (2025) 135:143–54. doi: 10.1016/j.anai.2025.05.016 40393554

[21] Rothenberg ME . Scientific journey to the first Fda-approved drug for eosinophilic esophagitis. J Allergy Clin Immunol. (2022) 150:1325–32. doi: 10.1016/j.jaci.2022.09.027 36209816 PMC9742179

[22] Patel SA . Real-world efficacy of dupilumab in four cases of paediatric-onset fibrostenotic eosinophilic esophagitis. Clin Exp Pharmacol Physiol. (2024) 51:e13903. doi: 10.1111/1440-1681.13903 38889994

[23] Braun C Grzywacz K Dal Soglio D Drouin E Chartier ME Dirks M . Dupilumab in eosinophilic gastrointestinal disorders with extraesophageal involvement: A pediatric case series and systematic review. J Investig Allergol Clin Immunol. (2025) 36(2):100–112. doi: 10.18176/jiaci.1098 41200751

[24] Sanchez-Ramirez A Garcia-Diaz A Garcia-Martinez de Bartolome R Garcia-Hidalgo MI Cilleruelo ML Roman-Riechmann E . Muscular and serosal eosinophilic gastritis and enteritis with eosinophilic ascites successfully treated with dupilumab. JPGN Rep. (2026) 7:132–6. doi: 10.1002/jpr3.70113 41695062 PMC12894091

[25] Visaggi P Bini L Castagnaro E Cairoli G Testi F Mitilini M . Efficacy of dupilumab for induction and maintenance of remission in patients with eosinophilic gastritis, enteritis, and colitis with concomitant eosinophilic esophagitis. Neurogastroenterol Motil. (2025) 37:e70177. doi: 10.1111/nmo.70177 41108548

[26] Piccirillo M Di Nardo G Pilozzi E Furio S Di Marino VP Pucinischi V . Dupilumab as a rescue therapy for steroid-dependent eosinophilic gastritis in a child unresponsive to elimination diet: A case report. Front Pediatr. (2026) 14:1755630. doi: 10.3389/fped.2026.1755630 42051955 PMC13111380

[27] Lim WK Wipperman MF Rothenberg ME Collins MH Hamon SC Chehade M . Dupilumab normalizes the eosinophilic esophagitis disease transcriptome. J Allergy Clin Immunol. (2026) 158:542–54. doi: 10.1016/j.jaci.2026.01.033 42031097

[28] Chakraborty S Sharma A Marella S Rizza CF O'Brien PA Ganesan V . Unique epithelial proliferative transcriptomic signature in proton pump inhibitor-responsive pediatric eosinophilic esophagitis. JCI Insight. (2025) 10(19):e178595. doi: 10.1172/jci.insight.178595 41059573 PMC12513502

[29] Lee CJ Dellon ES . Real-world efficacy of dupilumab in severe, treatment-refractory, and fibrostenotic patients with eosinophilic esophagitis. Clin Gastroenterol Hepatol. (2024) 22:252–8. doi: 10.1016/j.cgh.2023.08.015 37660770

[30] Laserna-Mendieta EJ Ubeda-Vargas V Amorena E Savarino EV Casabona-Frances S Coletta M . Dupilumab for eosinophilic esophagitis in adolescents and adults: Real-world outcomes across different dosing regimens from the Eoe Connect Registry. Am J Gastroenterol. (2026). doi: 10.14309/ajg.0000000000004088 42335360

[31] Kleuskens MTA Haasnoot ML Garssen J Bredenoord AJ van Esch B Redegeld FA . Transcriptomic profiling of the acute mucosal response to local food injections in adults with eosinophilic esophagitis. J Allergy Clin Immunol. (2024) 153:780–92. doi: 10.1016/j.jaci.2023.10.027 37972740

[32] Bedell A Taft T Craven MR Guadagnoli L Hirano I Gonsalves N . Impact on health-related quality of life in adults with eosinophilic gastritis and gastroenteritis: A qualitative assessment. Dig Dis Sci. (2018) 63:1148–57. doi: 10.1007/s10620-018-4978-7 29476289 PMC6701628

[33] Vidal Gonzalez A Lopez Alcazar S Escudero Tornero R Vergara Barria FA Amat Sanchez JA Maseda Pedrero R . Upadacitinib in children under 12 years with dupilumab-refractory severe atopic dermatitis: A real-world case series. Clin Exp Dermatol. (2026) 51:1482–5. doi: 10.1093/ced/llag143 41885167

[34] Sia T Bacchus L Kumar A Fikri L Solecki R Herath R . Upadacitinib for patients with eosinophilic esophagitis refractory to dupilumab. Allergol Int. (2026) 75:338–40. doi: 10.1016/j.alit.2025.11.010 41419361

