# Supplementary material for: The efficacy and safety of dupilumab therapy in pediatric patients with refractory eosinophilic duodenal bulb ulcer: a retrospective cohort study
Source: Front Immunol. 2026 Oct 27;17:1953390. doi: 10.3389/fimmu.2026.1953390 (PMC13748424; doi:10.3389/fimmu.2026.1953390)
Supplement: Supplementary file 1 [file DataSheet1.docx]

***Supplemental Figures***

Supplemental Figure 1. Panels A1–A2 through H1–H2 show paired endoscopic images of the duodenal bulb for Cases 1–8, obtained before and after 12 weeks of dupilumab treatment.

Supplemental Figure 2. Panels A1–A2 through E1–E2 show paired endoscopic images of the duodenal bulb for Cases 9–13, obtained before and after 12 weeks of dupilumab treatment. Note: Panel B2 shows the duodenal bulb endoscopic image of Case 10 obtained at 36 weeks of dupilumab therapy, as the 12-week endoscopic image was unavailable.

Figure 3. Endoscopic images and histological findings in patients with recurrent disease. Panels A1–A2 to C1–C2 display paired endoscopic images and high-power histological findings (magnification, 400×) of the duodenal bulb from Case 1, Case 6, and Case 8 at 24 weeks of additional dupilumab treatment following discontinuation of proton pump inhibitor therapy.
